# Iron Metabolism of the Skeletal Muscle and Neurodegeneration

**DOI:** 10.3389/fnins.2019.00165

**Published:** 2019-03-15

**Authors:** Malgorzata Halon-Golabek, Andzelika Borkowska, Anna Herman-Antosiewicz, Jedrzej Antosiewicz

**Affiliations:** ^1^Department of Physiotherapy, Faculty of Health Sciences, Medical University of Gdańsk, Gdańsk, Poland; ^2^Department of Bioenergetics and Physiology of Exercise, Faculty of Health Sciences, Medical University of Gdańsk, Gdańsk, Poland; ^3^Department of Medical Biology and Genetics, Faculty of Biology, University of Gdańsk, Gdańsk, Poland; ^4^Department of Biochemistry, Gdańsk University of Physical Education and Sport, Gdańsk, Poland

**Keywords:** myokine, iron, insulin signaling, ALS, skeletal muscle, neurodegeneration, Akt, JNK

## Abstract

Recent studies clearly indicate that the endocrine function of the skeletal muscle is essential for a long and healthy life. Regular exercise, which has been shown to stimulate the release of myokines, lowers the risk of many diseases, including Alzheimer’s and Parkinson’s disease, emphasizing the role of skeletal muscle in proper functioning of other tissues. In addition, exercise increases insulin sensitivity, which may also impact iron metabolism. Even though the role of iron in neurodegeneration is well established, the exact mechanisms of iron toxicity are not known. Interestingly, exercise has been shown to modulate iron metabolism, mainly by reducing body iron stores. Insulin signaling and iron metabolism are interconnected, as high tissue iron stores are associated with insulin resistance, and conversely, impaired insulin signaling may lead to iron accumulation in an affected tissue. Excess iron accumulation in tissue triggers iron-dependent oxidative stress. Further, iron overload in the skeletal muscle not only negatively affects muscle contractility but also might impact its endocrine function, thus possibly affecting the clinical outcome of diseases, including neurodegenerative diseases. In this review, we discuss possible mechanisms of iron dependent oxidative stress in skeletal muscle, its impact on muscle mass and endocrine function, as well as on neurodegeneration processes.

## Introduction

Excess of iron in any tissue may induce oxidative stress and impair tissue function. In the skeletal muscle, oxidative stress not only causes muscle damage but also negatively impacts its endocrine function. The skeletal muscle is a source of myokines, which are cytokines produced and released by skeletal muscle capable of exerting protective effects on other tissues, including the neuronal tissue (Besse-Patin et al., 2014; Dai et al., 2018; Liu et al., 2018). This is supported by the observation that regular exercise, which pronouncedly increases myokine biosynthesis, reduces the risk of various diseases, including Parkinson’s disease (PD) and Alzheimer’s disease (AD) (Chen et al., 2005; Santos-Lozano et al., 2016). Conversely, disruption of balance between muscle protein synthesis and degradation, resulting from a wide variety of conditions, including cancer, immobilization (or disuse), denervation, or iron overload, can lead to oxidative stress dependent skeletal muscle atrophy and impairment of myokine synthesis (Tisdale, 2004; Argiles et al., 2014). Although muscle function is widely studied in terms of adaptive changes induced by exercise, or atrophy induced by some morbidities, much less is known about its possible illness-related role as an endocrine tissue, and about the interconnections between oxidative stress and myokine production. This topic will probably represent one of the hot new areas in the study of pathomechanisms of neurodegeneration and other diseases.

Iron overload is a known contributor to multiple degenerative diseases, including liver fibrosis, heart attack, and cancer (Stevens et al., 1994; Klipstein-Grobusch et al., 1999; Ong and Farooqui, 2005). Importantly, excess iron accumulation in the brain is linked to neurodegenerative disorders (Bartzokis et al., 1999, 2000). Some neurodegenerative diseases are associated with the failure of muscle function (Busse et al., 2008). However, little is known about the link between iron accumulation in the muscle and neurodegeneration. Some interesting results come from studies on amyotrophic lateral sclerosis (ALS), a neurodegenerative disease characterized by a selective loss of motor neurons (Gajowiak et al., 2015). These findings, as well as current knowledge about iron metabolism in the skeletal muscle and its possible influence on neurodegenerative diseases, will be discussed in the current review.

## Endocrine Function of the Skeletal Muscle and Neurodegeneration

In recent years, the skeletal muscle has been recognized as a secretory organ that releases appreciable amounts of circulating proteins, called myokines. Currently, we know that the skeletal muscle produces several hundreds of peptides classified as myokines, and muscle contraction stimulates their release (Henningsen et al., 2010; Huh, 2018). Considering that the skeletal muscle represents the largest organ of the human body, (the muscles constitute approximately 40% of total body mass), its role in the regulation of metabolic processes *via* myokines appears to be very important. Myokines can act as autocrine, paracrine, or endocrine stimuli. Thus, they may affect different organs and tissues, e.g., the brain, bone, adipocyte tissue, heart artery, and many others (Giudice and Taylor, 2017). For instance, the myokines interleukin (IL) IL-6 and IL-10, released from the muscle during exercise or under ischemia, exert powerful local and systemic anti-inflammatory effects. Furthermore, IL-10 has been shown to provide cardio- and neuroprotection, mediated by the activation of anti-apoptotic protein kinase B (PKB or Akt) (Sharma et al., 2011; Cai et al., 2013). Physical activity induces central and peripheral production of neurotrophins, such as brain-derived neurotrophic factor (BDNF), glial cell line-derived neurotrophic factor (GDNF), neurotrophin-3 (NT-3), and neurotrophin-4 (NT-4). They support neural survival, growth, synaptic plasticity, and neuromuscular junctions (Zoladz and Pilc, 2010). In addition, myokines, such as myostatin, irisin, IL-15, IL-6, leukemia inhibitory factor (LIF), or apelin, play a major role in processes associated with regulation of hypertrophic muscle growth and myogenesis (Munoz-Canoves et al., 2013).

Limited data are available on the effect of oxidative stress on the biosynthesis of myokines, where myostatin is one of the examples. It is a member of transforming growth factor beta superfamily and negatively regulates muscle growth. The myostatin/follistatin ratio is significantly higher in ALS in comparison to control patients, and is positively correlated with muscle degeneration (Tasca et al., 2016). Oxidative stress has been shown to increase myostatin synthesis (Enoki et al., 2016) and, conversely, myostatin increases the production of reactive oxygen species (ROS) by NADPH oxidase in C2C12 cells (Sriram et al., 2011). Expression of myostatin is downregulated by regular exercise (Jones et al., 2004; Kim et al., 2005; Louis et al., 2007). Interestingly, compared with a sedentary ALS animal, swim training of ALS mouse significantly lowers oxidative stress and delays body weight reduction (Flis et al., 2018). In addition, it has been shown that swim training sustains the motor function and increases the ALS mouse life span by about 25 days. This beneficial effect is one of the most important therapeutic achievements in the strategy of ALS treatment. What is more, the analysis of muscle phenotype revealed maintenance of the fast phenotype in fast-twitch muscles, delayed spinal motoneuron death, and preserved astrocyte and oligodendrocyte populations in ALS spinal cord (Deforges et al., 2009). Recent data have shown that swimming exercise not only extends life span in mouse model of ALS, but also maintains the grip strength in ALS mice, lowers cholesterol content, and raises the caveolin-1 protein level in the skeletal muscle crude mitochondrial fraction. Moreover, higher activity of COX enzyme in swimming animals seems to be a marker of respiratory chain function improvement (Flis et al., 2018). However, the role of myokines in protective effects of swimming training on ALS development has not been studied.

**FIGURE 1:**
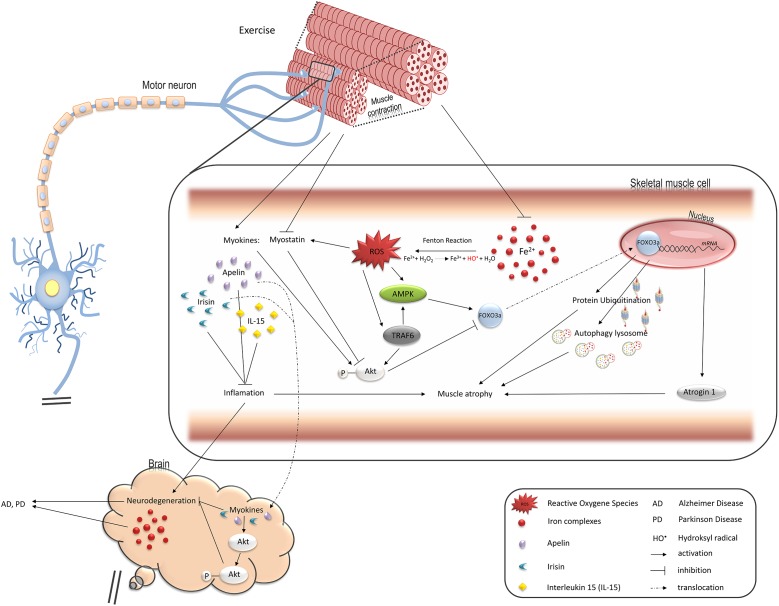
Pathways regulated by oxidative stress induced by iron overload in muscle cells and leading to muscle atrophy, changes in endocrine functions, and contributing to neurodegeneration. FOXO3a transcription factor upregulates two main protein degradation pathways, ubiquitin-proteasome and autophagy-lysosome, both involved in muscle atrophy (see details in the text). Iron-mediated ROS elevation inhibits activity of FOXO3a negative regulator, Akt, and stimulates its positive regulator, AMPK. TRAF6 ubiquitin ligase is also involved in stimulation of muscle atrophy mediated by FOXO3a as well as inflammatory response and might be activated by ROS. Ferrous iron-induced ROS have been shown to activate TRAF6 in hepatic macrophages. ROS also induce production of myostatin which leads to muscle atrophy. On the other hand, exercise downregulates myostatin while such myokines as apelin or IL15 are increased and stimulate Akt in skeletal muscle and neuronal tissue thus protect against muscle and neurons atrophy. Loss of muscle mass thus, reduction in their endocrine functions may accelerate neurodegeneration and degeneration of motor neurons promotes muscle atrophy.

The role of myostatin inhibitors as potential therapeutics for muscle-wasting diseases and muscle weakness in human and animals has been widely explored. Several myostatin inhibitors, including myostatin antibodies, anti-myostatin peptibody, activin A antibody, soluble (decoy) forms of soluble activin receptor type IIB (ActRIIB-Fc), anti-myostatin adnectin, and ActRIIB antibody have been tested in pre-clinical and clinical trials in the last decade. These inhibitors have currently progressed into clinical development in several indications, mainly sarcopenia, early recovery after surgery, and cachexia. Myostatin inhibitors for the treatment of muscular dystrophy are also being tested in early clinical trials (Saitoh et al., 2017). There are many papers showing positive effects of myostatin inhibitors on animal models with different types of muscle disorders (Holzbaur et al., 2006; Ohsawa et al., 2006; Morine et al., 2010). It has been demonstrated that treatment of an ALS mouse with myostatin inhibitor, ActRIIB-Fc, results in a delay in the onset of weakness, increases body weight and grip strength, and enlarges muscle size when applied either in a pre-symptomatic animal or after symptom onset (Morrison et al., 2009). Surprisingly, in an animal denervation model, myostatin inhibition is not effective against atrophy. By contrast, ActRIIB-Fc treatment protects immobilized mice against the loss of muscle mass (MacDonald et al., 2014). Myostatin is thought to disrupt the balance between protein synthesis and protein degradation in healthy skeletal muscle by inhibiting Akt kinase, which regulates the muscle mass by inhibiting protein degradation and promoting protein synthesis (Morissette et al., 2009; Trendelenburg et al., 2009; Glass, 2010; Figure 1). The most common, myostatin-dependent, signaling pathways involved in muscle myogenesis and protein synthesis/degradation include: Akt/mTOR/mTORC/p70s6K; Akt/FOXO/Atrogin-1, MURF-1; Akt/GSK-3β/cyclinD1 (Elkina et al., 2011; Rodriguez et al., 2014).

Contrary to myostatin, other myokines, e.g., apelin and IL-15, exert a protective effect against oxidative stress and skeletal muscle atrophy.

Apelin is a peptide which is an endogenous ligand for the apelin receptor (APJ) (Tatemoto et al., 1998). The apelin/APJ system has several important functions in the body, such as blood pressure regulation, cardiac contractility, immunity, glucose metabolism, water homeostasis, cell proliferation, angiogenesis, and neuroprotection (Wu et al., 2017). According to research carried out in recent years, apelin is considered to be a myokine (Besse-Patin et al., 2014; Son et al., 2018; Vinel et al., 2018). Vinel et al. (2018) demonstrated in their *in vitro* and *in vivo* studies that apelin reverses age-related sarcopenia. During aging, apelin synthesis in skeletal muscle is reduced and plasma apelin levels decrease. Conversely, aged mice, supplemented with a daily injection of apelin or overexpressing apelin, exhibited improved muscle capacities and myofiber hypertrophy (Vinel et al., 2018). A number of studies demonstrated that apelin mediates neuroprotection in *in vivo* and *in vitro* models (Kasai et al., 2011; Cheng et al., 2012; Zou et al., 2016; Ishimaru et al., 2017). Moreover, epidemiological and clinical studies reported that physical activity could reduce the risk of developing of PD and AD (Bhalsing et al., 2018; Zhang et al., 2018). Interestingly, apelin stimulates endothelial nitric oxide release that plays a role in inhibition of amyloid beta (Aβ) production, synaptic plasticity, and Aβ clearance in brain (Masoumi et al., 2018). In cells treated with 6-hydroxydopamine, which imitates dopaminergic neurotoxic conditions in PD, pretreatment with apelin-13 significantly decreased the level of intracellular ROS. In heart, apelin protects from ROS-dependent damage or cardiac hypertrophy (Zeng et al., 2009; Foussal et al., 2010). Apelin pretreatment decreased the generation of ROS and malonaldehyde content as well as lactate dehydrogenase leakage in myocardial cells from neonatal rats under hypoxia/re-oxygenation. Furthermore, apelin enhanced superoxide dismutase activity and phosphorylation of extracellular signal-regulated kinase 1/2 and Akt after hypoxia/re-oxygenation (Zeng et al., 2009). In a mouse model of ALS, mRNA levels of apelin and its receptor were significantly lower in spinal cord of the *G93A hmSOD1* mouse than those in the wild-type mouse. Immunohistochemical analysis revealed a reduced number of motor neurons and activation of microglial cells in transgenic *G93A hmSOD1* apelin-deficient mouse, indicating that apelin deficiency pathologically accelerates the progression of disease. Besides, apelin enhanced the protective effect of VEGF on hydrogen peroxide (H_2_O_2_)-induced neuronal death in primary neurons (Kasai et al., 2011).

Skeletal muscle tissue releases high amounts of IL-15, which is reported to increase transiently, immediately following resistance (Riechman et al., 2004) and aerobic exercise (Tamura et al., 2011). In the mice muscle and serum, IL-15 protein levels decline progressively with advanced age (Quinn et al., 2010). Further, Yalcin et al. (2018) demonstrated that the level of IL-15 is significantly lower in patients with sarcopenia compared to non-sarcopenic old people. A study conducted on a C2C12 muscle cell line presented the protective effects of IL-15 against H_2_O_2_-iduced oxidative stress. Pre-incubation with IL-15 reduced the intracellular creatine kinase and lactate dehydrogenase activities and decreased the ROS overproduction in H_2_O_2_ exposured myoblasts (Li et al., 2014).

Irisin a novel myokine, which is secreted following proteolytic cleavage of its precursor fibronectin type III domain containing 5 (FNDC5). Irisin plays a role in metabolic diseases, aging, inflammation, and neurogenesis (Mahgoub et al., 2018). Physical activity increases irisin level in plasma (Jedrychowski et al., 2015). During AD, the level of FNDC5 /irisin was decreased in hippocamp and cerebrospinal fluid. Knockdown of FNDC5/irisin in the brain impairs long-term potentiation and novel object recognition memory in mice. Conversely, boosting brain levels of FNDC5/irisin rescues synaptic plasticity and memory in AD mouse models. Peripheral overexpression of FNDC5/irisin rescues memory impairment, whereas blockade of either peripheral or brain FNDC5/irisin attenuates the neuroprotective actions of physical exercise on synaptic plasticity and memory in AD mice. Irisin reduced ischemia-induced neuronal injury, and significantly suppressed the levels of nitrotyrosine, superoxide anion, and 4-hydroxynonenal in peri-infarct brain tissues. Mice administration with irisin increased Akt and ERK1/2 phosphorylation, while blockade of Akt and ERK1/2 by specific inhibitors reduced the neuroprotective effects of this compound. Finally, the exercised mice injected with irisin neutralizing antibody displayed more severe neuronal injury than the exercised mice injected with control IgG (Li et al., 2017). In obese patients and in chronic diseases, such as type I and type II diabetes or chronic kidney disease, the level of irisin is lower than in healthy normal subjects (Wen et al., 2013; Ebert et al., 2014; Belviranli et al., 2016; Lu et al., 2016; Shelbaya et al., 2017). The above-mentioned cases, as well as other chronic diseases, are accompanied by chronic inflammation.

## Dysregulation of Iron Metabolism and Skeletal Muscle Atrophy

Loss of muscle mass is caused by an imbalance between protein synthesis and muscle fiber degradation. Two main degradation pathways can be hyperactivated during muscle dystrophy: the ubiquitin-proteasome and autophagy-lysosome systems. These two pathways require ATP and are believed to serve separate functions. Proteasomes degrade myofibrillar and short-lived proteins (Solomon and Goldberg, 1996; Clarke et al., 2007; Fielitz et al., 2007; Cohen et al., 2009), whereas autophagy-lysosomes remove long-lived proteins and organelles (Levine and Kroemer, 2008; Mizushima et al., 2008).

The ubiquitin-proteasome system relies on a cascade of enzymatic reactions that culminate in the labeling of substrate proteins with ubiquitin chains, for degradation by the 26S proteasome. E3 ubiquitin ligases confer substrate specificity and play a crucial role in this system. Two E3 ubiquitin ligases are essential for the development of skeletal muscle atrophy: muscle atrophy F-box (MAFbx)/atrogin-1 and muscle RING finger-1 (MuRF1). They are responsible for the selection and ubiquitination of myofibrillar proteins for subsequent proteosomal degradation (Bodine et al., 2001; Gomes et al., 2001).

The autophagy-lysosomal pathway involves sequestration of substrates within vacuoles called autophagosomes. These vacuoles subsequently fuse with lysosomes, and the cargo is hydrolyzed by lysosomal hydrolases. This process is controlled by autophagy-specific gene products, including Beclin 1. Crucial stages of the pathway rely on the transfer of small ubiquitin-like molecules (LC3 and others) from the conjugation system to the membranes, to allow their growth into double-membrane autophagosomes that engulf portions of the cytoplasm (Kabeya et al., 2000; Mizushima et al., 2004).

Interestingly, both pathways are upregulated during atrophy by Forkhead box (FOX) O3a, which regulates the transcription of genes coding for atrogin-1, MuRF1, LC3B, and its homolog Gabarap 1, as well as Beclin 1 (Sandri et al., 2004; Mammucari et al., 2007; Zhao et al., 2007). Transcriptional activity of FOXO3a is regulated by posttranslational modifications. Regulation by Akt has been most extensively investigated; the protein phosphorylates FOXO3a on Thr32 and Ser253, leading to its cytosolic retention by 14-3-3 (Brunet et al., 1999). Consequently, factors that activate Akt, such as insulin or the growth factor/phosphatidylinositide 3-kinase (PI3K) pathway, cause FOXO3a inactivation and prevent the synthesis of proteins involved in muscle atrophy. By contrast, phosphorylation of Ser413/588 of FOXO3a by AMP-activated protein kinase, a protein that becomes activated during energy deficit (exercise, hypoxia, or nutritional stress), leads to its activation and, subsequently, the induction of protein degradation pathways (Sandri, 2010; Sanchez et al., 2012).

The relationship between muscle iron metabolism and muscle atrophy with age or disease is unclear; however, recent reports have shed some light on these processes. Ikeda et al. (2016) showed that iron administration results in a decrease of skeletal muscle mass in mouse. The molecular mechanism of this phenomenon involved the induction of oxidative stress and inhibition of the Akt-FOXO3a pathway, hence, upregulation of atrogin-1 and MuRF1. Silencing of FOXO3a expression in C2C12 myotube cells or application of ROS scavenger, TEMPOL, suppress iron-induced expression of atrogin-1 and MuRF1, and prevent cell atrophy (Ikeda et al., 2016). Furthermore, Huang et al. (2013) demonstrated that mouse fed a high-iron diet exhibits elevated AMP-activated protein kinase activity and impaired insulin signaling in the skeletal muscle and liver. These effects are abrogated by co-treatment with *N*-acetyl cysteine (Huang et al., 2013).

Another regulator of the degradation pathways is tumor necrosis factor receptor-associated factor (TRAF6). It induces the expression of muscle-specific E3 ubiquitin ligases and autophagy-related molecules in the skeletal muscle on denervation and in Lewis lung carcinoma tumor-bearing mouse (Paul et al., 2010). It is worth noting that iron might stimulate this signaling pathway which was shown in hepatic macrophages (Zhong et al., 2012). Thus, iron accumulation in the skeletal muscle may play an underlying role in skeletal muscle atrophy (Figure 1).

## Iron Accumulation in the Skeletal Muscle

Under physiological conditions, most iron is stored in the liver, spleen, and bone marrow; however, a high amount of iron has also been detected in the skeletal muscle. It has been determined that the total amount of stored iron in the skeletal muscle is comparable with that in the liver in healthy individuals (Torrance et al., 1968). Furthermore, muscle storage of iron can increase, e.g., in individuals with iron overload.

Iron metabolism seems to be tightly controlled. It is not entirely clear why under some conditions iron accumulates in the skeletal muscle and/or other tissues as well. It has been demonstrated that diet rich in highly bioavailable forms of iron promotes high iron stores, whereas foods containing phytate and other natural iron chelators reduce these stores. Conversely, under some pathological conditions, excessive iron accumulation is observed regardless of the diet. For example, in an animal model of ALS, the amount of iron and iron storage proteins, ferritin L and ferritin H, is elevated in the skeletal muscles and neurons (Jeong et al., 2009; Halon-Golabek et al., 2018). Skeletal muscle iron accumulation has also been observed after immobilization (Kondo et al., 1992). Further, hepatic iron content significantly increases after 2 weeks of a high-fructose diet (Ackerman et al., 2005). These data clearly indicate that tissue iron accumulation is not always associated with the consumption of food with high-iron content (Tsuchiya et al., 2013) but, rather, with impaired tissue iron metabolism. The mechanism of iron transport into a cell is well understood; however, the changes in iron metabolism that are responsible for excess iron accumulation are not fully known.

Iron overload can negatively affect skeletal muscle function, as it can induce oxidative stress (Schafer et al., 1981). Intracellular ROS formation is strongly associated with the amount of free iron. Lowering the levels of catalytic free iron in a cell by using chelators always results in reduced ROS formation and changes the composition of free radical species. For example, formation of the hydroxyl radical is iron-dependent.

It is not clear why increased iron stores correlate with enhanced iron-dependent oxidative stress since iron, stored mostly in ferritin, does not stimulate ROS formation. Despite this, a positive correlation between oxidative DNA damage and body iron stores has been observed (Barollo et al., 2004; Sullivan, 2004). Iron may affect the clinical course of diseases associated with the pathological disorders of the muscle. We have recently shown that in a transgenic rat bearing the *G93A hmSOD1* gene (an animal model of familial ALS), iron levels in the muscle increased with the development of disease, and that was accompanied by increased oxidative stress (Halon et al., 2014; Halon-Golabek et al., 2018). Taken together, similarly to the brain, liver, and some other tissues, under certain conditions, the skeletal muscle may accumulate too much iron, which contributes to ROS formation.

## Stress and Iron Signaling

**FIGURE 2:**
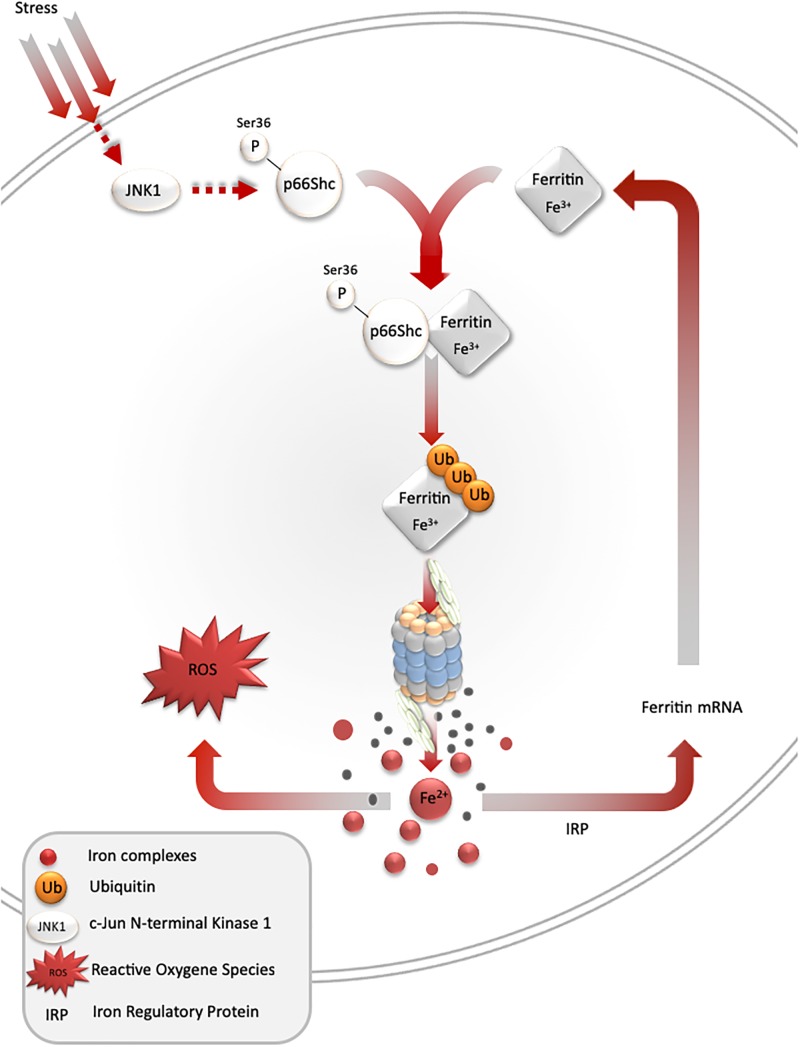
Stress-mediated ferritin degradation leads to increase in iron-dependent ROS formation. Overexpression of *SOD1 G93A* leads to JNK and p66Shc activation, ferritin ubiquitination, and degradation by proteasome. As a result, LIP and iron-dependent ROS formation increase. In addition, iron augments ferritin synthesis in order to overcome iron toxicity.

Under stress conditions, numerous signaling pathways are activated within a cell, which may lead to an adaptive response to such conditions. One of such pathways is mediated by stress-activated protein kinases, and results in ferritin degradation and release of free iron [the so-called labile iron pool (LIP)]. In cell culture models, c-jun terminal kinase (JNK-1), a stress-activated protein kinase, together with p66Shc adaptor protein, mediates ferritin degradation by the proteasome (Antosiewicz et al., 2006, 2007; Borkowska et al., 2011). Ferritin is a protein that binds iron atoms and stores them in a “safe” (non-reactive) form. One molecule of ferritin can bind up to 4500 iron atoms in the form of ferric iron (Fe^3+^), creating a mineral core, in which the iron is stored in complex with phosphate. However, ferritin may be a source of free iron if it undergoes proteasomal or lysosomal degradation. Recent studies indicate that ferritin can be also degraded by autophagy mediated by nuclear receptor co-activator 4 (Philpott et al., 2017). These and some other studies clearly show that ferritin iron is not a “safe” form of iron, as it can be liberated and subsequently stimulate iron-dependent cell damage (Sullivan, 2004; Figure 2).

Despite the ability to induce cell damage, iron is also a physiological signaling molecule. Its signaling properties are associated with ROS formation. ROS can oxidize specific amino acids in proteins and thus modulate their activity; e.g., iron-responsive protein 2 in the presence of high iron levels undergoes site-specific oxidation, which targets this protein for proteolytic degradation (Iwai et al., 1998). Cysteine is an amino acid with a very high affinity for chelatable iron. Interestingly, cysteine oxidation by H_2_O_2_ is often not possible in the absence of iron. That is because of the high p*K*a of sulfydryl groups of most cysteine residues in proteins (approximately 8.5). Only cysteine thiolate anion (Cys-S-) is vulnerable to oxidation by H_2_O_2_. Thus, the amount of iron released during ferritin degradation may greatly impact cellular response to stress.

One of the most important signaling activities of iron is associated with its interaction with iron-responsive element-binding proteins, which can upregulate ferritin gene expression. Increases in ferritin L and H levels lead to the sequestration of free iron, and reduction of iron-dependent ROS formation, lowering tissue damage. Such a scenario has been observed in an ischemic heart, in which ischemic preconditioning induces iron-dependent upregulation of ferritin, protecting the heart during full ischemia (Chevion et al., 2008). Certainly, the amount of iron liberated during ferritin degradation will determine tissue responses to stress. For example, in a mouse model of iron overload, elevated levels of iron in the tibialis anterior muscle and a fourfold increase in ferritin light chains were observed. These changes were accompanied by elevated markers of oxidative stress; significant reduction in the fast-twitch (extensor digitorum longus) and slow-twitch (soleus) muscles mass; and decreased exercise capacity (Reardon and Allen, 2009). Moreover, cytotoxicity of the tumor necrosis factor was augmented by iron and significantly reduced by iron chelators on cell line model (Warren et al., 1993).

## Insulin Sensitivity and Iron Accumulation in the Skeletal Muscle

Insulin or growth factors/PI3K/Akt/FOXO3a is another important signaling pathway that is affected by stress. Insulin resistance is observed in numerous pathological conditions and is associated with the impaired Akt/FOXO3a signaling pathway. Insulin and growth factors-mediated activation of Akt is facilitated by its membrane recruitment upon interaction with phosphatidylinositol 3,4,5-trisphosphate synthesized by PI3K. After membrane anchoring, Akt is phosphorylated at Ser308 and Ser473 by phosphoinositide-dependent kinase 1 and mammalian target of rapamycin complex 2, respectively. Upon phosphorylation, Akt translocates from the plasma membrane to intracellular compartments, including the nucleus, where it phosphorylates a range of substrates (Song et al., 2005). One of these substrates is transcriptional factor FOXO3a, phosphorylated by Akt at Thr32, Ser253, and Ser315. FOXO3a phosphorylation leads to its exclusion from the nucleus and reduction of its DNA-binding activity. Stress conditions may activate JNK, which phosphorylates Ser574 of FOXO3a, antagonizes the Akt signaling pathway, and promotes nuclear translocation and transcriptional activity of FOXO3a (Sunayama et al., 2005; Wang et al., 2012). FOXO3a plays an important role in the upregulation of genes associated with oxidative stress resistance, including catalase and MnSOD genes.

Recently, it has been demonstrated that *Caenorhabditis elegans* DAF-16, an ortholog of the FOXO family of transcription factors, regulates iron metabolism by increasing the expression of ferritin H (Ackerman and Gems, 2012). We confirmed this observation using a mammalian cell line, demonstrating that an increase in FOXO3a activity leads to upregulation of ferritin protein levels in cells (Halon-Golabek et al., 2018). This observation was confirmed in the skeletal muscle of transgenic animals expressing *SOD1 G93A* in which Akt activity reduction, FOXO3a activity increase, and upregulation of ferritin protein and catalase activity were observed (Halon-Golabek et al., 2018).

Thus, impairment in insulin signaling increases FOXO3a-mediated induction of antioxidant proteins, such as catalase, MnSOD, ferritin, and increases cellular resistance to oxidative stress. Under some conditions, increasing ferritin H protein levels is sufficient to augment cell antioxidant potential. For example, in a cell culture model, overexpression of ferritin H leads to 50% reduction of LIP and increased resistance to oxidative challenge (Cozzi et al., 2000). In the short term, such changes are beneficial for the cell; however, in the longer term, such conditions can disturb iron metabolism. Accordingly, overexpression of ferritin H leads to reduced iron-dependent signaling and induction of an iron-deficiency phenotype. These changes are manifested by a fivefold increase in the activity of iron-responsive element-binding proteins; 2.5-fold increase of transferrin receptor levels; and 1.8-fold increase in iron-transferrin uptake (Cozzi et al., 2000). Hence, it can be expected that during chronic stress, upregulation of ferritin may lead to reduced LIP levels.

**FIGURE 3:**
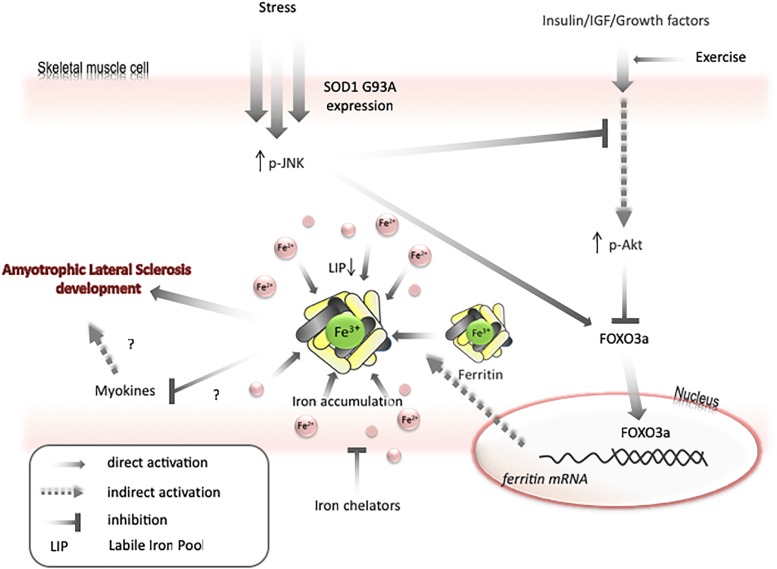
Model for mechanisms of iron accumulation in muscle. Expression of *SOD1 G93A* or other stresses lead to activation of JNK and inactivation of Akt. It results in activation of FOXO3a transcriptional factor. FOXO3a upregulates ferritin H expression that can cause decrease in labile iron pool and upregulate iron transport into a cell. Iron accumulation may negatively affect myokines synthesis and increase motor neuron vulnerability to degeneration. Exercise can prevent AKT inactivation and iron chelators can diminish skeletal muscle accumulation.

Transfer of some labile iron to ferritin may generate iron-deficiency phenotype, even if the total amount of iron in a cell is unchanged. Under such conditions, the cell will continue to import iron until LIP returns to the usual level. Therefore, as mentioned above, both Akt and JNK kinases can be involved in iron metabolism by modulating FOXO3a/ferritin activity. Further, it has been shown on cell lines that JNK-mediated ferritin degradation is accompanied by increase in LIP and iron-dependent ROS formation (Antosiewicz et al., 2007; Borkowska et al., 2011). JNK activation and a decrease in Akt activity have been observed in ALS humans and animals (Kim and Choi, 2015). Interestingly, decreases in Akt levels, upregulation of ferritin L, and decreases in ferritin H levels are observed in the muscle of ALS pre-symptomatic animals. These changes are accompanied by increased levels of oxidative stress markers, possibly because of impaired iron metabolism. Hence, it can be speculated that LIP transiently increases before the first symptoms of disease in the muscle of ALS animals, unblocking the translation of ferritin and activating FOXO3a, to augment the transcription of ferritin genes. In later phases of disease, upregulation of ferritin may cause iron accumulation and iron-dependent induction of oxidative stress (Figure 3). Interestingly, impairment insulin signaling and iron accumulation have also been observed in the brain of PD and AD (Aviles-Olmos et al., 2013; Calsolaro and Edison, 2016; Rani et al., 2016; Apostolakis and Kypraiou, 2017). Insulin receptors are found in the basal ganglia and substantia nigra and growing evidences are suggesting that insulin plays an essential regulating role in neuronal survival and growth, dopaminergic transmission, and maintenance of synapses (Bassil et al., 2014). Thus, there are some evidences that patients with type 2 diabetes (DMT2) have an increased risk of developing PD and share similar dysregulated pathways suggesting common underlying pathological mechanisms (Dunn et al., 2014). In the early stage of the DMT2, patients develop insulin resistance, leading to a variety of detrimental effects on metabolism and inflammation. Accumulating evidence suggests that similar dysregulation of glucose and energy metabolism seems to be an early event in the pathogenesis of sporadic PD, indicating that the insulin signaling pathway may potentially be a novel target for disease modification (Athauda and Foltynie, 2016). There is a fundamental question, whether insulin resistance occurs as a cause or a consequence of neurodegeneration. Substantial evidence implicates that loss of Akt control (an important downstream target of insulin signaling pathway) is involved in DMT2 and AD (Griffin et al., 2005). There are strong evidences that an altered Akt signaling pathway could be a component of PD neurodegeneration (Greene et al., 2011). Reduction in phosphorylated Akt kinase was observed in post mortem studies of PD patients (Malagelada et al., 2008; Timmons et al., 2009). In contrary to skeletal muscle, the interdependence between insulin signaling and iron metabolism in neuronal tissue has not been studied. We can only speculate that changes in insulin signaling manifested by the inhibition of PI3K/Akt/FOXO3a signaling pathway may trigger changes in iron metabolism which will lead to brain iron accumulation and iron-dependent oxidative stress.

## The Role of Iron in Pathomechanism of ALS

Amyotrophic lateral sclerosis, also known as Lou Gehrig’s disease, is a progressive, usually fatal, neurodegenerative disease in human adulthood. It is caused by the degeneration of motor neurons in the spinal cord, i.e., the nerve cells in the central nervous system that control voluntary muscle movement. ALS is characterized by escalating muscle weakness, spasticity, and atrophy as both the upper and lower motor neurons degenerate (Rowland, 2001; Lino et al., 2002; Gajowiak et al., 2016). Recent studies indicate that the nerve cells and non-neuronal cells may both play a decisive role in the onset of disease. Transgenic mice harboring *SOD1 G93A* gene recapitulates the symptoms of human ALS and are considered as an animal model of the disease. Interestingly, expression of the *SOD1* variant exclusively in the motor neurons results in no apparent pathology or motor deficit suggesting that accumulation of the *hm SOD1* gene in neurons is not a critical factor for the onset of the disease (Lino et al., 2002). Furthermore, studies involving the animal model indicate that restricted expression of the mutated *SOD1* gene in astrocytes, neurofilaments, or oligodendrocytes is not sufficient to cause motor neuron degeneration *in vivo*. Surprisingly, these animals fail to develop the disease, which confirms the notion that the expression of *SOD1 G93A* in other cell types is critical for disease initiation, or that other factors beside the presence of mutated *SOD1* gene are essential for the onset of disease (Gong et al., 2000; Pramatarova et al., 2001; Yamanaka et al., 2008). Many scientific reports indicate oxidative stress as an initiating factor in the onset of the disease. In a transgenic mouse model, selective expression of mutated *SOD1 G93A* (Dobrowolny et al., 2008) or *G37R* (Wong and Martin, 2010) exclusively in the skeletal muscle demonstrated progressive muscle atrophy, associated with a significant reduction in muscle strength, alterations in the contractile apparatus-sarcomere and sarcotubular system disorganization, and mitochondrial dysfunction, mimicking a pathologic phenotype consistent with ALS. These data demonstrate that skeletal muscle is a primary target of *SOD1(G93A)*-mediated toxicity and disclose the molecular mechanism whereby oxidative stress triggers muscle atrophy, where human SOD1 has a causal role in ALS and motor neuron degeneration explaining their selective vulnerability (Dobrowolny et al., 2008; Wong and Martin, 2010).

Thanks to recent advances in genetics, we now know that ALS is associated with mutations in at least 20 genes, coding for proteins related to cell functions as diverse as RNA metabolism (*TARDBP*, *FUS/TLS*, *Senataxin*, *Ataxin2*, *HNRNPA2/B1*, *ELP3*, *HNRNPA1*), vesicle trafficking (*Alsin*, *FIG4*, *OPTN*, *VABP*, *CHMP2B*), and proteasomal function (*UBQLN2*, *VCP*) (White and Sreedharan, 2016). The GGGGCC hexanucleotide repeat expansion in gene *C9orf72* is the most numerous genetic variant of ALS, which together with genetic modifications in *SOD1*, *TARDBP*, *FUS*, *Antaxin*, or *OPTN* proteins, is extensively explored, in the ALS context.

The analyses of disorders in iron metabolism in ALS patients, in most cases, concern patients in whom the etiology of the disease is unknown. By contrast, in animal models, rodents of mutated *hm SOD1* gene (most commonly *SOD1 G93A* and *SOD1 G37R*) predominate. In ALS patients, iron accumulation was observed in the spinal cord and cerebrospinal fluid (Kasarskis et al., 1995). MRI technic is a useful method to detect iron deposits in the brain in ALS patients. There are some evidence showing iron aggregates in the precentral gyrus’s gray matter, which shift toward higher MRI scores after 6 months (Ignjatovic et al., 2013). In postmortem, histopathological analysis of a brains, Perls’ DAB staining, revealed abundant cells containing intracellular iron and occasional extracellular iron deposits in the motor cortex of ALS patients. The intracellular iron was observed in microglia, which was accompanied by a higher level of ferritin (Kwan et al., 2012). In addition, patients with ALS have marked changes in the iron biochemical parameters measured in the blood. Studies have shown increased serum ferritin levels, transferrin saturation coefficient, and decreased transferrin in ALS patients, which could reflect a general increase in iron stores or be a consequence of on-going muscle degeneration (Goodall et al., 2008; Qureshi et al., 2008; Nadjar et al., 2012; Hu et al., 2016). In addition, elevated serum ferritin levels have been associated with reduced survival. Patients with high level serum ferritin had a shorter survival time compared to those with low level serum ferritin (618 days versus 921 days). However, increased serum ferritin levels are not specifically indicative of increased body iron storage. Systemic inflammation can also be associated with increased serum ferritin, especially during the end-stage of disease, and positively correlates with bronchial congestion in patients with respiratory muscle weakness (Nadjar et al., 2012).

Amyotrophic lateral sclerosis animal models allow for more accurate investigation of molecular mechanisms of iron accumulation in the nervous system or muscles. Jeong et al. (2009) proved iron accumulation in the spinal cord of *SOD1G37R* transgenic mice at 12 months of age. In the cell bodies of the large ventral horn motor neurons in the spinal cord, the level of cytoplasmic iron inclusions (measured by ferrozine) was increased by 56% in *SOD1G37R* mice, compared with age-matched wild-type controls (Jeong et al., 2009). On the other hand, changes in iron metabolism were observed in skeletal muscles of *SOD1 G93A* transgenic rats. Changes in expression of the iron metabolism proteins (H-ferritin and ferroportin) in skeletal muscles were already observed at the pre-symptomatic stage in *SOD1G93A* rats. During disease progression, the level of ferritin H significantly increased and was accompanied by iron accumulation (Halon et al., 2014).

Information on iron metabolism disorders in ALS models other than those expressing mutations in the *SOD1* gene is poor. In 2006, the gene coding for transactive response DNA binding protein 43 kDa (TDP-43), an RNA/DNA binding protein, was implicated in ALS as the major component of ubiquitinated inclusions (Neumann et al., 2006). TDP-43 is cleaved, hyperphosphorylated, ubiquitinated, or mis-localized in the cytoplasm in the form of insoluble inclusions. Guam-ALS patients presented higher iron levels and lower zinc levels in brain (Yasui et al., 1993). ALS and ALS/PD-dementia patients in Guam also present TDP-43 inclusions as a secondary pathology. In this paper, it has also been shown that the TDP-43 is a consistent component of the ubiquitinated inclusions in sporadic ALS and Guam ALS, but TDP-43 inclusions are absent or scarce in SOD1-familial ALS (Maekawa et al., 2009). On the contrary, evidence shows that TDP-43 was expressed by astrocytes and microglial cells in the spinal cord of *hmSOD1(G93A)* transgenic mice. In addition, the expression of phosphorylated and truncated TDP-43 increased in the spinal cord of ALS mice compared with age-matched non-transgenic (Tg) (Cai et al., 2015; Jeon et al., 2018). Furthermore, the serum iron concentration and expression of transferrin (a homeostasis-related iron protein) in the SP were increased relative to non-Tg. The protein expression level of heme oxygenase 1 related to oxidative stress was increased in the spinal cord of *hmSOD1(G93A)* Tg relative to non-Tg (Cai et al., 2015). In *TDP-43 A315T* transgenic mice, dysregulation of ion metal was noticed. However, only the levels of zinc, copper, and manganese were increased in the spinal cords significantly. The level of iron has not changed significantly (Dang et al., 2014). Many ALS patients (∼36–51%) also exhibit cognitive impairment, with frontotemporal lobar degeneration (FTLD) in about 20%. There is some probability that patients with FTLD might develop ALS. Pathologically, FTLD includes multiple subtypes, including FTLD-TDP-43, FTLD-FUS (fused in sarcoma/translocated in liposarcoma), or FTLD-Tau (Guerrero et al., 2016). Postmortem research conducted on human brains showed that a significant increase of iron deposition was observed in the claustrum, caudate nucleus, globus pallidus, thalamus, and subthalamic nucleus of the FTLD-FUS and FTLD-TDP groups, while in the ALS one, the iron increase was only observed in the caudate and the subthalamic nuclei. FTLD is often linked to mutations in *FUS* and *TDP43or* to an expanded *C9orf72* repeat.

There are no data on skeletal muscle iron metabolism in ALS models other than ALS-SOD. What is interesting, in patients with FTLD, the mutation in hemochromatosis gene *(H63D HFE)* genotype was found. Further, increased iron uptake, and gradual iron accumulation in tissues and organs have been reported in patients with a mutation of the *HFE* gene, which encodes a protein responsible for the control of iron absorption by the intestinal epithelial cells. Other studies indicate that the H63D mutation within the gene is a contributing factor in the development of ALS (Wang et al., 2004; Sutedja et al., 2007). Interestingly, studies show increased prevalence of *HFE* gene mutations in individuals with AD (Percy et al., 2008). Conversely, studies on animal models demonstrated that double transgenic generated mouse line (*SOD1/H67D*) carrying the *H67D HFE* (homolog of human H63D) and *SOD1(G93A)* mutations had a shorter survival and accelerated disease progression comparing to *SOD1 G93A* mouse. This correlated with decreased transferrin receptor and a significant increase in ferritin L expression-indicators of iron status (L-ferritin expression in double transgenic mice was 86 and 79% higher than SOD1 mice at 90 and 110 days in the double transgenic and SOD1 mice starting at 90 days pre-symptomatic stage), indicating for dysregulation of iron homeostasis in these mice. What is more, compared to SOD1 mice, double transgenic mice performed significantly worse on both forelimb and hindlimb grip strength beginning at the age of disease onset (106 days) until the end of the test (127 days), suggesting an accelerated disease progression in these mice (Nandar et al., 2014).

In support of the role of iron in the development of ALS, it has been reported that the use of iron chelator salicylaldehyde isonicotinoyl hydrazone significantly delays the onset of disease and increases the life span (by approximately 5 weeks) depending on the dosage, which resulted in a five- to sixfold decrease in the number of accumulating iron cells in the spinal cord. These studies clearly indicate that the disturbance of iron homeostasis, or rather its excessive accumulation in central nervous system, leads to the progression of these animals’ disease (Jeong et al., 2009). In addition, treatment with another chelator, deferoxamine, does not alter the permeability of the blood–spinal cord barrier to IgG, hemoglobin, or hemosiderin. Instead, it significantly reduced early accumulation of free iron, which was accompanied by a delayed onset of disease in comparison with warfarin- or saline-treated patients. These data suggest that deferoxamine prevents early iron accumulation in the spinal cord but does not exert a direct anti-inflammatory effect (Zhong et al., 2009; Winkler et al., 2014). Similarly to deferoxamine, VK-28 and monoamine oxidase inhibitor (M30) are brain-permeable iron chelators that exhibit neuroprotective and neurorestorative activities in NCS-34 motor neuron cells (Kupershmidt et al., 2009; Wang et al., 2011). Treatment with VK-28 and M30 delays the disease onset, prolongs the lifespan, and reduces spinal cord motor neuron loss in a mouse model of ALS. Furthermore, iron chelators attenuate the elevated level of iron and the expression of transferrin receptor; decrease the production of free oxygen radicals; and suppress microglial and astrocytic activation in the spinal cord of the *SOD1 G93A* mouse. Furthermore, iron chelators decrease toxic aggregation of the transcriptional regulator TDP-43, decrease the levels of proapoptotic Bax, and increased the levels of antiapoptotic protein Bcl-2 (Wang et al., 2011).

The notion of complex etiology of neurodegenerative disorders led to the discovery of a brain-permeable, non-toxic compound with antiapoptotic and iron-chelating properties—VAR10303. When co-administered with a high-calorie/energy diet to *SOD1 G93A* mouse, VAR10303 prolonged life span for about 10%, improved motor performance, and attenuated iron accumulation and motor neuron loss in the animal spinal cord (Golko-Perez et al., 2016). This slight extension of the life span in the context of chronic disease seems to have a remarkable meaning and what is far more important, VAR treatment significantly increased stride length distance—a useful gait footprint parameter in mouse model of ALS.

Recently published results indicate that conservative iron chelation—deferiprone (i.e., chelation with low risk of iron depletion), in a murine preclinical model and pilot clinical trial in *SOD1 G86R* mice, increased the mean life span compared with placebo. This corresponded to a 56% extension in survival (13 days) from disease onset in a female mouse compared to the vehicle group (defined as the peak in the body weight). What is more interesting, an interaction between dose and sex was observed, namely, the required dose was higher in males than in females. Treatment with deferiprone led to a decrease in iron accumulation not only in the murine model *SOD1 G86R*, but also in ALS patients where a significant decrease in iron concentration was observed in the cervical spinal cord, medula oblongata and motor cortex after 12 months of treatment, but the first visible signs were already seen after 3 months. All patients had a slight elevation in urine iron levels and presented a decreased level of 8-OHdG in the cerebrospinal fluid after 9 months of treatment. Summing up, iron chelators might be promising agents for clinical treatment, as there is a strong evidence indicating the role of iron dysregulation in neuronal cell death the pathophysiology of neuromuscular diseases. The presented and study demonstrates the safety of conservative iron chelation in ALS, as even at a low-dose levels, deferiprone can cross membranes, decrease iron accumulation, may re-enter the captured iron into extracellular transferrin, and then spread the iron throughout the body, thus avoiding anemia (Moreau et al., 2018).

## Conclusion

Neurodegenerative diseases are associated with insulin resistance and iron accumulation not only in the central nervous system but also in the muscle. The exact mechanism of iron accumulation in the skeletal muscle and neuronal tissue is yet unknown; however, there is evidence for the involvement of impaired insulin and growth factor/PI3K/Akt/FOXO3a signaling pathways. This accumulation may lead to increased production of ROS and oxidative stress, which may impair endocrine function of the skeletal muscle and, indirectly, the function of other organs. Hence, under such conditions, the neuronal tissue suffers not only from iron-induced oxidative stress but also from a reduced exposure to trophic factors derived from the skeletal muscle. Thus, it is possible that changes in the iron metabolism might constitute a trigger of a disease, as opposed to being caused by the disease. This notion is supported by observation that changes in iron metabolism are observed before the first symptoms of disease in ALS rats. The protective effects of iron chelators in many models of neurodegeneration confirm the important role of this metal in the pathomechanism of these diseases and indicate that the chelators could have some therapeutic value. Exercise training which is known to increase insulin sensitivity and modulate iron metabolism could be recommended as a preventive approach.

## Author Contributions

MH-G, AB, AH-A, and JA conceptualized the study and wrote the manuscript. AH-A and JA reviewed the manuscript.

## Conflict of Interest Statement

The authors declare that the research was conducted in the absence of any commercial or financial relationships that could be construed as a potential conflict of interest.

## References

[B1] Ackerman D. Gems D. (2012). Insulin/IGF-1 and hypoxia signaling act in concert to regulate iron homeostasis in Caenorhabditis elegans. *PLoS Genet.* 8:e1002498. 10.1371/journal.pgen.1002498 22396654 PMC3291539

[B2] Ackerman Z. Oron-Herman M. Grozovski M. Rosenthal T. Pappo O. Link G. (2005). Fructose-induced fatty liver disease: hepatic effects of blood pressure and plasma triglyceride reduction. *Hypertension* 45 1012–1018. 10.1161/01.HYP.0000164570.20420.67 15824194

[B3] Antosiewicz J. Herman-Antosiewicz A. Marynowski S. W. Singh S. V. (2006). c-Jun NH(2)-terminal kinase signaling axis regulates diallyl trisulfide-induced generation of reactive oxygen species and cell cycle arrest in human prostate cancer cells. *Cancer Res.* 66 5379–5386. 10.1158/0008-5472.CAN-06-0356 16707465

[B4] Antosiewicz J. Ziolkowski W. Kaczor J. J. Herman-Antosiewicz A. (2007). Tumor necrosis factor-alpha-induced reactive oxygen species formation is mediated by JNK1-dependent ferritin degradation and elevation of labile iron pool. *Free Radic. Biol. Med.* 43 265–270. 10.1016/j.freeradbiomed.2007.04.023 17603935

[B5] Apostolakis S. Kypraiou A. M. (2017). Iron in neurodegenerative disorders: being in the wrong place at the wrong time? *Rev. Neurosci.* 28 893–911. 10.1515/revneuro-2017-0020 28792913

[B6] Argiles J. M. Fontes-Oliveira C. C. Toledo M. Lopez-Soriano F. J. Busquets S. (2014). Cachexia: a problem of energetic inefficiency. *J. Cachexia Sarcopenia Muscle* 5 279–286. 10.1007/s13539-014-0154-x 25118829 PMC4248416

[B7] Athauda D. Foltynie T. (2016). Insulin resistance and Parkinson’s disease: a new target for disease modification? *Prog. Neurobiol.* 14 98–120. 10.1016/j.pneurobio.2016.10.001 27713036

[B8] Aviles-Olmos I. Limousin P. Lees A. Foltynie T. (2013). Parkinson’s disease, insulin resistance and novel agents of neuroprotection. *Brain* 136 374–384. 10.1093/brain/aws009 22344583

[B9] Barollo M. D’Inca R. Scarpa M. Medici V. Cardin R. Fries W. (2004). Effects of iron deprivation or chelation on DNA damage in experimental colitis. *Int. J. Colorectal. Dis.* 19 461–466. 10.1007/s00384-004-0588-2 15067556

[B10] Bartzokis G. Cummings J. L. Markham C. H. Marmarelis P. Z. Treciokas L. J. Tishler T. A. (1999). MRI evaluation of brain iron in earlier- and later-onset Parkinson’s disease and normal subjects. *Magn. Reson. Imaging* 17 213–222. 10.1016/S0730-725X(98)00155-6 10215476

[B11] Bartzokis G. Sultzer D. Cummings J. Holt L. E. Hance D. B. Henderson V. W. (2000). In vivo evaluation of brain iron in Alzheimer disease using magnetic resonance imaging. *Arch. Gen. Psychiatry* 57 47–53. 10.1001/archpsyc.57.1.47 10632232

[B12] Bassil F. Fernagut P. O. Bezard E. Meissner W. G. (2014). Insulin, IGF-1 and GLP-1 signaling in neurodegenerative disorders: targets for disease modification? *Prog. Neurobiol.* 118 1–18. 10.1016/j.pneurobio.2014.02.005 24582776

[B13] Belviranli M. Okudan N. Celik F. (2016). Association of circulating irisin with insulin resistance and oxidative stress in obese women. *Horm. Metab. Res.* 48 653–657. 10.1055/s-0042-116155 27632149

[B14] Besse-Patin A. Montastier E. Vinel C. Castan-Laurell I. Louche K. Dray C. (2014). Effect of endurance training on skeletal muscle myokine expression in obese men: identification of apelin as a novel myokine. *Int. J. Obes.* 38 707–713. 10.1038/ijo.2013.158 23979219

[B15] Bhalsing K. S. Abbas M. M. Tan L. C. S. (2018). Role of physical activity in Parkinson’s disease. *Ann. Indian Acad. Neurol.* 21 242–249. 10.4103/aian.AIAN_169_18 30532351 PMC6238554

[B16] Bodine S. C. Latres E. Baumhueter S. Lai V. K. Nunez L. Clarke B. A. (2001). Identification of ubiquitin ligases required for skeletal muscle atrophy. *Science* 294 1704–1708. 10.1126/science.1065874 11679633

[B17] Borkowska A. Sielicka-Dudzin A. Herman-Antosiewicz A. Halon M. Wozniak M. Antosiewicz J. (2011). P66Shc mediated ferritin degradation–a novel mechanism of ROS formation. *Free Radic. Biol. Med.* 51 658–663. 10.1016/j.freeradbiomed.2011.04.045 21616139

[B18] Brunet A. Bonni A. Zigmond M. J. Lin M. Z. Juo P. Hu L. S. (1999). Akt promotes cell survival by phosphorylating and inhibiting a Forkhead transcription factor. *Cell* 96 857–868. 10.1016/S0092-8674(00)80595-4 10102273

[B19] Busse M. E. Hughes G. Wiles C. M. Rosser A. E. (2008). Use of hand-held dynamometry in the evaluation of lower limb muscle strength in people with Huntington’s disease. *J. Neurol.* 255 1534–1540. 10.1007/s00415-008-0964-x 19005627

[B20] Cai M. Lee K. W. Choi S. M. Yang E. J. (2015). TDP-43 modification in the hSOD1(G93A) amyotrophic lateral sclerosis mouse model. *Neurol. Res.* 37 253–262. 10.1179/1743132814Y.0000000443 25213598

[B21] Cai Z. Luo W. Zhan H. Semenza G. L. (2013). Hypoxia-inducible factor 1 is required for remote ischemic preconditioning of the heart. *Proc. Natl. Acad. Sci. U.S.A.* 110 17462–17467. 10.1073/pnas.1317158110 24101519 PMC3808664

[B22] Calsolaro V. Edison P. (2016). Alterations in glucose metabolism in Alzheimer’s disease. *Recent Pat. Endocr. Metab. Immune. Drug Discov.* 10 31–39. 10.2174/1872214810666160615102809 27306508

[B23] Chen H. Zhang S. M. Schwarzschild M. A. Hernan M. A. Ascherio A. (2005). Physical activity and the risk of Parkinson disease. *Neurology* 64 664–669. 10.1212/01.WNL.0000151960.28687.93 15728289

[B24] Cheng B. Chen J. Bai B. Xin Q. (2012). Neuroprotection of apelin and its signaling pathway. *Peptides* 37 171–173. 10.1016/j.peptides.2012.07.012 22820556

[B25] Chevion M. Leibowitz S. Aye N. N. Novogrodsky O. Singer A. Avizemer O. (2008). Heart protection by ischemic preconditioning: a novel pathway initiated by iron and mediated by ferritin. *J. Mol. Cell Cardiol.* 45 839–845. 10.1016/j.yjmcc.2008.08.011 18817783

[B26] Clarke B. A. Drujan D. Willis M. S. Murphy L. O. Corpina R. A. Burova E. (2007). The E3 Ligase MuRF1 degrades myosin heavy chain protein in dexamethasone-treated skeletal muscle. *Cell Metab.* 6 376–385. 10.1016/j.cmet.2007.09.009 17983583

[B27] Cohen S. Brault J. J. Gygi S. P. Glass D. J. Valenzuela D. M. Gartner C. (2009). During muscle atrophy, thick, but not thin, filament components are degraded by MuRF1-dependent ubiquitylation. *J. Cell Biol.* 185 1083–1095. 10.1083/jcb.200901052 19506036 PMC2711608

[B28] Cozzi A. Corsi B. Levi S. Santambrogio P. Albertini A. Arosio P. (2000). Overexpression of wild type and mutated human ferritin H-chain in HeLa cells: in vivo role of ferritin ferroxidase activity. *J. Biol. Chem.* 275 25122–25129. 10.1074/jbc.M003797200 10833524

[B29] Dai T. T. Wang B. Xiao Z. Y. You Y. Tian S. W. (2018). Apelin-13 upregulates BDNF against chronic stress-induced depression-like phenotypes by ameliorating HPA axis and hippocampal glucocorticoid receptor dysfunctions. *Neuroscience* 390 151–159. 10.1016/j.neuroscience.2018.08.018 30170158

[B30] Dang T. N. Lim N. K. Grubman A. Li Q. X. Volitakis I. White A. R. (2014). Increased metal content in the TDP-43(A315T) transgenic mouse model of frontotemporal lobar degeneration and amyotrophic lateral sclerosis. *Front. Aging Neurosci.* 6:15. 10.3389/fnagi.2014.00015 24575040 PMC3920072

[B31] Deforges S. Branchu J. Biondi O. Grondard C. Pariset C. Lecolle S. (2009). Motoneuron survival is promoted by specific exercise in a mouse model of amyotrophic lateral sclerosis. *J. Physiol.* 587 3561–3572. 10.1113/jphysiol.2009.169748 19491245 PMC2742281

[B32] Dobrowolny G. Aucello M. Rizzuto E. Beccafico S. Mammucari C. Bonconpagni S. (2008). Skeletal muscle is a primary target of SOD1G93A-mediated toxicity. *Cell Metab.* 8 425–436. 10.1016/j.cmet.2008.09.002 19046573

[B33] Dunn L. Allen G. F. Mamais A. Ling H. Li A. Duberley K. E. (2014). Dysregulation of glucose metabolism is an early event in sporadic Parkinson’s disease. *Neurobiol. Aging* 35 1111–1115. 10.1016/j.neurobiolaging.2013.11.001 24300239 PMC3969149

[B34] Ebert T. Focke D. Petroff D. Wurst U. Richter J. Bachmann A. (2014). Serum levels of the myokine irisin in relation to metabolic and renal function. *Eur. J. Endocrinol.* 170 501–506. 10.1530/EJE-13-1053 24399249

[B35] Elkina Y. von Haehling S. Anker S. D. Springer J. (2011). The role of myostatin in muscle wasting: an overview. *J. Cachexia Sarcopenia Muscle* 2 143–151. 10.1007/s13539-011-0035-5 21966641 PMC3177043

[B36] Enoki Y. Watanabe H. Arake R. Sugimoto R. Imafuku T. Tominaga Y. (2016). Indoxyl sulfate potentiates skeletal muscle atrophy by inducing the oxidative stress-mediated expression of myostatin and atrogin-1. *Sci. Rep.* 6:32084. 10.1038/srep32084 27549031 PMC4994088

[B37] Fielitz J. Kim M. S. Shelton J. M. Latif S. Spencer J. A. Glass D. J. (2007). Myosin accumulation and striated muscle myopathy result from the loss of muscle RING finger 1 and 3. *J. Clin. Invest.* 117 2486–2495. 10.1172/JCI32827 17786241 PMC1957544

[B38] Flis D. J. Dzik K. Kaczor J. J. Halon-Golabek M. Antosiewicz J. Wieckowski M. R. (2018). Swim training modulates skeletal muscle energy metabolism, oxidative stress, and mitochondrial cholesterol content in amyotrophic lateral sclerosis mice. *Oxid. Med. Cell Longev.* 2018:5940748. 10.1155/2018/5940748 29849903 PMC5924974

[B39] Foussal C. Lairez O. Calise D. Pathak A. Guilbeau-Frugier C. Valet P. (2010). Activation of catalase by apelin prevents oxidative stress-linked cardiac hypertrophy. *FEBS Lett.* 584 2363–2370. 10.1016/j.febslet.2010.04.025 20398658

[B40] Gajowiak A. Stys A. Starzynski R. R. Bednarz A. Lenartowicz M. Staron R. (2015). Mice overexpressing both non-mutated human SOD1 and mutated SOD1(G93A) genes: a competent experimental model for studying iron metabolism in amyotrophic lateral sclerosis. *Front. Mol. Neurosci.* 8:82. 10.3389/fnmol.2015.00082 26778957 PMC4701970

[B41] Gajowiak A. Stys A. Starzynski R. R. Staron R. Lipinski P. (2016). Misregulation of iron homeostasis in amyotrophic lateral sclerosis. *Postepy Hig. Med. Dosw.* 70 709–721. 10.5604/17322693.1208036 27356602

[B42] Giudice J. Taylor J. M. (2017). Muscle as a paracrine and endocrine organ. *Curr. Opin. Pharmacol.* 34 49–55. 10.1016/j.coph.2017.05.005 28605657 PMC5808999

[B43] Glass D. J. (2010). Signaling pathways perturbing muscle mass. *Curr. Opin. Clin. Nutr. Metab. Care* 13 225–229. 10.1097/MCO.0b013e32833862df 20397318

[B44] Golko-Perez S. Amit T. Youdim M. B. Weinreb O. (2016). Beneficial effects of multitarget iron chelator on central nervous system and gastrocnemius muscle in SOD1(G93A) Transgenic ALS Mice. *J. Mol. Neurosci.* 59 504–510. 10.1007/s12031-016-0763-2 27173029

[B45] Gomes M. D. Lecker S. H. Jagoe R. T. Navon A. Goldberg A. L. (2001). Atrogin-1, a muscle-specific F-box protein highly expressed during muscle atrophy. *Proc. Natl. Acad. Sci. U.S.A.* 98 14440–14445. 10.1073/pnas.251541198 11717410 PMC64700

[B46] Gong Y. H. Parsadanian A. S. Andreeva A. Snider W. D. Elliott J. L. (2000). Restricted expression of G86R Cu/Zn superoxide dismutase in astrocytes results in astrocytosis but does not cause motoneuron degeneration. *J. Neurosci.* 20 660–665. 10.1523/JNEUROSCI.20-02-00660.2000 10632595 PMC6772423

[B47] Goodall E. F. Haque M. S. Morrison K. E. (2008). Increased serum ferritin levels in amyotrophic lateral sclerosis (ALS) patients. *J. Neurol.* 255 1652–1656. 10.1007/s00415-008-0945-0 18677636

[B48] Greene L. A. Levy O. Malagelada C. (2011). Akt as a victim, villain and potential hero in Parkinson’s disease pathophysiology and treatment. *Cell Mol. Neurobiol.* 31 969–978. 10.1007/s10571-011-9671-8 21547489 PMC3678379

[B49] Griffin R. J. Moloney A. Kelliher M. Johnston J. A. Ravid R. Dockery P. (2005). Activation of Akt/PKB, increased phosphorylation of Akt substrates and loss and altered distribution of Akt and PTEN are features of Alzheimer’s disease pathology. *J. Neurochem.* 93 105–117. 10.1111/j.1471-4159.2004.02949.x 15773910

[B50] Guerrero E. N. Wang H. Mitra J. Hegde P. M. Stowell S. E. Liachko N. F. (2016). TDP-43/FUS in motor neuron disease: Complexity and challenges. *Progr. Neurobiol.* 14 78–97. 10.1016/j.pneurobio.2016.09.004 27693252 PMC5101148

[B51] Halon M. Kaczor J. J. Ziolkowski W. Flis D. J. Borkowska A. Popowska U. (2014). Changes in skeletal muscle iron metabolism outpace amyotrophic lateral sclerosis onset in transgenic rats bearing the G93A hmSOD1 gene mutation. *Free Radic. Res.* 48 1363–1370. 10.3109/10715762.2014.955484 25175826

[B52] Halon-Golabek M. Borkowska A. Kaczor J. J. Ziolkowski W. Flis D. J. Knap N. (2018). hmSOD1 gene mutation-induced disturbance in iron metabolism is mediated by impairment of Akt signalling pathway. *J. Cachexia Sarcopenia Muscle* 9 557–569. 10.1002/jcsm.12283 29380557 PMC5989766

[B53] Henningsen J. Rigbolt K. T. Blagoev B. Pedersen B. K. Kratchmarova I. (2010). Dynamics of the skeletal muscle secretome during myoblast differentiation. *Mol. Cell. Proteomics* 9 2482–2496. 10.1074/mcp.M110.002113 20631206 PMC2984231

[B54] Holzbaur E. L. Howland D. S. Weber N. Wallace K. She Y. Kwak S. (2006). Myostatin inhibition slows muscle atrophy in rodent models of amyotrophic lateral sclerosis. *Neurobiol. Dis.* 23 697–707. 10.1016/j.nbd.2006.05.009 16837207

[B55] Hu X. Yang Y. Su J. Yao C. (2016). Meta-analysis of the relationship between amyotrophic lateral sclerosis and susceptibility to serum ferritin level elevation. *Neurosciences* 21 120–125. 10.17712/nsj.2016.2.20150482 27094521 PMC5107265

[B56] Huang J. Simcox J. Mitchell T. C. Jones D. Cox J. Luo B. (2013). Iron regulates glucose homeostasis in liver and muscle via AMP-activated protein kinase in mice. *FASEB J.* 27 2845–2854. 10.1096/fj.12-216929 23515442 PMC3688748

[B57] Huh J. Y. (2018). The role of exercise-induced myokines in regulating metabolism. *Arch. Pharm. Res.* 41 14–29. 10.1007/s12272-017-0994-y 29177585

[B58] Ignjatovic A. Stevic Z. Lavrnic S. Dakovic M. Bacic G. (2013). Brain iron MRI: a biomarker for amyotrophic lateral sclerosis. *J. Magn. Reson. Imaging* 38 1472–1479. 10.1002/jmri.24121 23564606

[B59] Ikeda Y. Imao M. Satoh A. Watanabe H. Hamano H. Horinouchi Y. (2016). Iron-induced skeletal muscle atrophy involves an Akt-forkhead box O3-E3 ubiquitin ligase-dependent pathway. *J. Trace Elem. Med. Biol.* 35 66–76. 10.1016/j.jtemb.2016.01.011 27049128

[B60] Ishimaru Y. Sumino A. Kajioka D. Shibagaki F. Yamamuro A. Yoshioka Y. (2017). Apelin protects against NMDA-induced retinal neuronal death via an APJ receptor by activating Akt and ERK1/2, and suppressing TNF-alpha expression in mice. *J. Pharmacol. Sci.* 133 34–41. 10.1016/j.jphs.2016.12.002 28087150

[B61] Iwai K. Drake S. K. Wehr N. B. Weissman A. M. LaVaute T. Minato N. (1998). Iron-dependent oxidation, ubiquitination, and degradation of iron regulatory protein 2: implications for degradation of oxidized proteins. *Proc. Natl. Acad. Sci. U.S.A.* 95 4924–4928. 10.1073/pnas.95.9.4924 9560204 PMC20189

[B62] Jedrychowski M. P. Wrann C. D. Paulo J. A. Gerber K. K. Szpyt J. Robinson M. M. (2015). Detection and quantitation of circulating human irisin by tandem mass spectrometry. *Cell Metab.* 22 734–740. 10.1016/j.cmet.2015.08.001 26278051 PMC4802359

[B63] Jeon G. S. Shim Y. M. Lee D. Y. Kim J. S. Kang M. Ahn S. H. (2018). Pathological Modification of TDP-43 in amyotrophic lateral sclerosis with SOD1 Mutations. *Mol. Neurobiol.* 10.1007/s12035-018-1218-2 [Epub ahead of print]. 29982983 PMC6394608

[B64] Jeong S. Y. Rathore K. I. Schulz K. Ponka P. Arosio P. David S. (2009). Dysregulation of iron homeostasis in the CNS contributes to disease progression in a mouse model of amyotrophic lateral sclerosis. *J. Neurosci.* 29 610–619. 10.1523/JNEUROSCI.5443-08.2009 19158288 PMC6665152

[B65] Jones S. W. Hill R. J. Krasney P. A. O’Conner B. Peirce N. Greenhaff P. L. (2004). Disuse atrophy and exercise rehabilitation in humans profoundly affects the expression of genes associated with the regulation of skeletal muscle mass. *FASEB J.* 18 1025–1027. 10.1096/fj.03-1228fje 15084522

[B66] Kabeya Y. Mizushima N. Ueno T. Yamamoto A. Kirisako T. Noda T. (2000). LC3, a mammalian homologue of yeast Apg8p, is localized in autophagosome membranes after processing. *EMBO J.* 19 5720–5728. 10.1093/emboj/19.21.5720 11060023 PMC305793

[B67] Kasai A. Kinjo T. Ishihara R. Sakai I. Ishimaru Y. Yoshioka Y. (2011). Apelin deficiency accelerates the progression of amyotrophic lateral sclerosis. *PLoS One* 6:e23968. 10.1371/journal.pone.0023968 21887354 PMC3161091

[B68] Kasarskis E. J. Tandon L. Lovell M. A. Ehmann W. D. (1995). Aluminum, calcium, and iron in the spinal cord of patients with sporadic amyotrophic lateral sclerosis using laser microprobe mass spectroscopy: a preliminary study. *J. Neurol. Sci.* 130 203–208. 10.1016/0022-510X(95)00037-3 8586987

[B69] Kim E. K. Choi E. J. (2015). Compromised MAPK signaling in human diseases: an update. *Arch. Toxicol.* 89 867–882. 10.1007/s00204-015-1472-2 25690731

[B70] Kim J. S. Cross J. M. Bamman M. M. (2005). Impact of resistance loading on myostatin expression and cell cycle regulation in young and older men and women. *Am. J. Physiol. Endocrinol. Metab.* 288 E1110–E1119. 10.1152/ajpendo.00464.2004 15644458

[B71] Klipstein-Grobusch K. Grobbee D. E. den Breeijen J. H. Boeing H. Hofman A. Witteman J. C. (1999). Dietary iron and risk of myocardial infarction in the Rotterdam Study. *Am. J. Epidemiol.* 149 421–428. 10.1093/oxfordjournals.aje.a009829 10067901

[B72] Kondo H. Miura M. Nakagaki I. Sasaki S. Itokawa Y. (1992). Trace element movement and oxidative stress in skeletal muscle atrophied by immobilization. *Am. J. Physiol.* 262 E583–E590. 10.1152/ajpendo.1992.262.5.E583 1590370

[B73] Kupershmidt L. Weinreb O. Amit T. Mandel S. Carri M. T. Youdim M. B. (2009). Neuroprotective and neuritogenic activities of novel multimodal iron-chelating drugs in motor-neuron-like NSC-34 cells and transgenic mouse model of amyotrophic lateral sclerosis. *FASEB J.* 23 3766–3779. 10.1096/fj.09-130047 19638399

[B74] Kwan J. Y. Jeong S. Y. Van Gelderen P. Deng H. X. Quezado M. M. Danielian L. E. (2012). Iron accumulation in deep cortical layers accounts for MRI signal abnormalities in ALS: correlating 7 tesla MRI and pathology. *PLoS One* 7:e35241. 10.1371/journal.pone.0035241 22529995 PMC3328441

[B75] Levine B. Kroemer G. (2008). Autophagy in the pathogenesis of disease. *Cell* 132 27–42. 10.1016/j.cell.2007.12.018 18191218 PMC2696814

[B76] Li D. J. Li Y. H. Yuan H. B. Qu L. F. Wang P. (2017). The novel exercise-induced hormone irisin protects against neuronal injury via activation of the Akt and ERK1/2 signaling pathways and contributes to the neuroprotection of physical exercise in cerebral ischemia. *Metab. Clin. Exp.* 68 31–42. 10.1016/j.metabol.2016.12.003 28183451

[B77] Li F. Li Y. Tang Y. Lin B. Kong X. Oladele O. A. (2014). Protective effect of myokine IL-15 against H2O2-mediated oxidative stress in skeletal muscle cells. *Mol. Biol. Rep.* 41 7715–7722. 10.1007/s11033-014-3665-9 25103021

[B78] Lino M. M. Schneider C. Caroni P. (2002). Accumulation of SOD1 mutants in postnatal motoneurons does not cause motoneuron pathology or motoneuron disease. *J. Neurosci.* 22 4825–4832. 10.1523/JNEUROSCI.22-12-04825.2002 12077179 PMC6757755

[B79] Liu D. R. Hu W. Chen G. Z. (2018). Apelin-12 exerts neuroprotective effect against ischemia-reperfusion injury by inhibiting JNK and P38MAPK signaling pathway in mouse. *Eur. Rev. Med. Pharmacol. Sci.* 22 3888–3895. 10.26355/eurrev_201806_15273 29949164

[B80] Louis E. Raue U. Yang Y. Jemiolo B. Trappe S. (2007). Time course of proteolytic, cytokine, and myostatin gene expression after acute exercise in human skeletal muscle. *J. Appl. Physiol.* 103 1744–1751. 10.1152/japplphysiol.00679.2007 17823296

[B81] Lu Y. Li H. Shen S. W. Shen Z. H. Xu M. Yang C. J. (2016). Swimming exercise increases serum irisin level and reduces body fat mass in high-fat-diet fed Wistar rats. *Lipids Health Dis.* 15:93. 10.1186/s12944-016-0263-y 27177924 PMC4866429

[B82] MacDonald E. M. Andres-Mateos E. Mejias R. Simmers J. L. Mi R. Park J. S. (2014). Denervation atrophy is independent from Akt and mTOR activation and is not rescued by myostatin inhibition. *Dis. Models Mech.* 7 471–481. 10.1242/dmm.014126 24504412 PMC3974457

[B83] Maekawa S. Leigh P. N. King A. Jones E. Steele J. C. Bodi I. (2009). TDP-43 is consistently co-localized with ubiquitinated inclusions in sporadic and Guam amyotrophic lateral sclerosis but not in familial amyotrophic lateral sclerosis with and without SOD1 mutations. *Neuropathology* 29 672–683. 10.1111/j.1440-1789.2009.01029.x 19496940

[B84] Mahgoub M. O. D’Souza C. Al Darmaki R. Baniyas M. Adeghate E. (2018). An update on the role of irisin in the regulation of endocrine and metabolic functions. *Peptides* 104 15–23. 10.1016/j.peptides.2018.03.018 29608940

[B85] Malagelada C. Jin Z. H. Greene L. A. (2008). RTP801 is induced in Parkinson’s disease and mediates neuron death by inhibiting Akt phosphorylation/activation. *J. Neurosci.* 28 14363–14371. 10.1523/JNEUROSCI.3928-08.2008 19118169 PMC3865436

[B86] Mammucari C. Milan G. Romanello V. Masiero E. Rudolf R. Del Piccolo P. (2007). FoxO3 controls autophagy in skeletal muscle in vivo. *Cell Metab.* 6 458–471. 10.1016/j.cmet.2007.11.001 18054315

[B87] Masoumi J. Abbasloui M. Parvan R. Mohammadnejad D. Pavon-Djavid G. Barzegari A. (2018). Apelin, a promising target for Alzheimer disease prevention and treatment. *Neuropeptides* 70 76–86. 10.1016/j.npep.2018.05.008 29807653

[B88] Mizushima N. Levine B. Cuervo A. M. Klionsky D. J. (2008). Autophagy fights disease through cellular self-digestion. *Nature* 451 1069–1075. 10.1038/nature06639 18305538 PMC2670399

[B89] Mizushima N. Yamamoto A. Matsui M. Yoshimori T. Ohsumi Y. (2004). In vivo analysis of autophagy in response to nutrient starvation using transgenic mice expressing a fluorescent autophagosome marker. *Mol. Biol. Cell* 15 1101–1111. 10.1091/mbc.e03-09-0704 14699058 PMC363084

[B90] Moreau C. Danel V. Devedjian J. C. Grolez G. Timmerman K. Laloux C. (2018). Could conservative iron chelation lead to neuroprotection in amyotrophic lateral sclerosis? *Antioxid. Redox Signal.* 29 742–748. 10.1089/ars.2017.7493 29287521 PMC6067092

[B91] Morine K. J. Bish L. T. Selsby J. T. Gazzara J. A. Pendrak K. Sleeper M. M. (2010). Activin IIB receptor blockade attenuates dystrophic pathology in a mouse model of Duchenne muscular dystrophy. *Muscle Nerve* 42 722–730. 10.1002/mus.21743 20730876 PMC4505731

[B92] Morissette M. R. Cook S. A. Buranasombati C. Rosenberg M. A. Rosenzweig A. (2009). Myostatin inhibits IGF-I-induced myotube hypertrophy through Akt. *Am. J. Physiol. Cell Physiol.* 297 C1124–C1132. 10.1152/ajpcell.00043.2009 19759331 PMC2777401

[B93] Morrison B. M. Lachey J. L. Warsing L. C. Ting B. L. Pullen A. E. Underwood K. W. (2009). A soluble activin type IIB receptor improves function in a mouse model of amyotrophic lateral sclerosis. *Exp. Neurol.* 217 258–268. 10.1016/j.expneurol.2009.02.017 19285073

[B94] Munoz-Canoves P. Scheele C. Pedersen B. K. Serrano A. L. (2013). Interleukin-6 myokine signaling in skeletal muscle: a double-edged sword? *FEBS J*. 280 4131–4148. 10.1111/febs.12338 23663276 PMC4163639

[B95] Nadjar Y. Gordon P. Corcia P. Bensimon G. Pieroni L. Meininger V. (2012). Elevated serum ferritin is associated with reduced survival in amyotrophic lateral sclerosis. *PLoS One* 7:e45034. 10.1371/journal.pone.0045034 23024788 PMC3443244

[B96] Nandar W. Neely E. B. Simmons Z. Connor J. R. (2014). H63D HFE genotype accelerates disease progression in animal models of amyotrophic lateral sclerosis. *Biochim. Biophys. Acta* 1842 2413–2426. 10.1016/j.bbadis.2014.09.016 25283820

[B97] Neumann M. Sampathu D. M. Kwong L. K. Truax A. C. Micsenyi M. C. Chou T. T. (2006). Ubiquitinated TDP-43 in frontotemporal lobar degeneration and amyotrophic lateral sclerosis. *Science* 314 130–133. 10.1126/science.1134108 17023659

[B98] Ohsawa Y. Hagiwara H. Nakatani M. Yasue A. Moriyama K. Murakami T. (2006). Muscular atrophy of caveolin-3-deficient mice is rescued by myostatin inhibition. *J. Clin. Invest.* 116 2924–2934. 10.1172/JCI28520 17039257 PMC1592547

[B99] Ong W. Y. Farooqui A. A. (2005). Iron, neuroinflammation, and Alzheimer’s disease. *J. Alzheimers Dis.* 8 183–200; discussion 209–215. 10.3233/JAD-2005-8211 16308487

[B100] Paul P. K. Gupta S. K. Bhatnagar S. Panguluri S. K. Darnay B. G. Choi Y. (2010). Targeted ablation of TRAF6 inhibits skeletal muscle wasting in mice. *J. Cell Biol.* 191 1395–1411. 10.1083/jcb.201006098 21187332 PMC3010064

[B101] Percy M. Moalem S. Garcia A. Somerville M. J. Hicks M. Andrews D. (2008). Involvement of ApoE E4 and H63D in sporadic Alzheimer’s disease in a folate-supplemented Ontario population. *J. Alzheimers Dis.* 14 69–84. 10.3233/JAD-2008-14107 18525129

[B102] Philpott C. C. Ryu M. S. Frey A. Patel S. (2017). Cytosolic iron chaperones: proteins delivering iron cofactors in the cytosol of mammalian cells. *J. Biol. Chem.* 292 12764–12771. 10.1074/jbc.R117.791962 28615454 PMC5546017

[B103] Pramatarova A. Laganiere J. Roussel J. Brisebois K. Rouleau G. A. (2001). Neuron-specific expression of mutant superoxide dismutase 1 in transgenic mice does not lead to motor impairment. *J. Neurosci.* 21 3369–3374. 10.1523/JNEUROSCI.21-10-03369.2001 11331366 PMC6762496

[B104] Quinn L. S. Anderson B. G. Strait-Bodey L. Wolden-Hanson T. (2010). Serum and muscle interleukin-15 levels decrease in aging mice: correlation with declines in soluble interleukin-15 receptor alpha expression. *Exp. Gerontol.* 45 106–112. 10.1016/j.exger.2009.10.012 19854259 PMC2814937

[B105] Qureshi M. Brown R. H. Jr. Rogers J. T. Cudkowicz M. E. (2008). Serum ferritin and metal levels as risk factors for amyotrophic lateral sclerosis. *Open Neurol. J.* 2 51–54. 10.2174/1874205X00802010051 19452011 PMC2627516

[B106] Rani V. Deshmukh R. Jaswal P. Kumar P. Bariwal J. (2016). Alzheimer’s disease: Is this a brain specific diabetic condition? *Physiol. Behav.* 164 259–267. 10.1016/j.physbeh.2016.05.041 27235734

[B107] Reardon T. F. Allen D. G. (2009). Iron injections in mice increase skeletal muscle iron content, induce oxidative stress and reduce exercise performance. *Exp. Physiol.* 94 720–730. 10.1113/expphysiol.2008.046045 19201785

[B108] Riechman S. E. Balasekaran G. Roth S. M. Ferrell R. E. (2004). Association of interleukin-15 protein and interleukin-15 receptor genetic variation with resistance exercise training responses. *J. Appl. Physiol.* 97 2214–2219. 10.1152/japplphysiol.00491.2004 15531573

[B109] Rodriguez J. Vernus B. Chelh I. Cassar-Malek I. Gabillard J. C. Hadj Sassi A. (2014). Myostatin and the skeletal muscle atrophy and hypertrophy signaling pathways. *Cell. Mol. Life Sci.* 71 4361–4371. 10.1007/s00018-014-1689-x 25080109 PMC11113773

[B110] Rowland L. P. (2001). How amyotrophic lateral sclerosis got its name: the clinical-pathologic genius of Jean-Martin Charcot. *Arch. Neurol.* 58 512–515. 10.1001/archneur.58.3.512 11255459

[B111] Saitoh M. Ishida J. Ebner N. Anker S. D. Springer J. Von Haehling S. (2017). Myostatin inhibitors as pharmacological treatment for muscle wasting and muscular dystrophy. *J. Cachexia Sarcopenia Muscle Clin. Rep.* 2:e00037 10.17987/jcsm-cr.v2i1.37

[B112] Sanchez A. M. Csibi A. Raibon A. Cornille K. Gay S. Bernardi H. (2012). AMPK promotes skeletal muscle autophagy through activation of forkhead FoxO3a and interaction with Ulk1. *J. Cell. Biochem.* 113 695–710. 10.1002/jcb.23399 22006269

[B113] Sandri M. (2010). Autophagy in skeletal muscle. *FEBS Lett.* 584 1411–1416. 10.1016/j.febslet.2010.01.056 20132819

[B114] Sandri M. Sandri C. Gilbert A. Skurk C. Calabria E. Picard A. (2004). Foxo transcription factors induce the atrophy-related ubiquitin ligase atrogin-1 and cause skeletal muscle atrophy. *Cell* 117 399–412. 10.1016/S0092-8674(04)00400-3 15109499 PMC3619734

[B115] Santos-Lozano A. Pareja-Galeano H. Sanchis-Gomar F. Quindos-Rubial M. Fiuza-Luces C. Cristi-Montero C. (2016). Physical activity and Alzheimer disease: a protective association. *Mayo Clin. Proc.* 91 999–1020. 10.1016/j.mayocp.2016.04.024 27492909

[B116] Schafer A. I. Cheron R. G. Dluhy R. Cooper B. Gleason R. E. Soeldner J. S. (1981). Clinical consequences of acquired transfusional iron overload in adults. *N. Engl. J. Med.* 304 319–324. 10.1056/NEJM198102053040603 6777701

[B117] Sharma S. Yang B. Xi X. Grotta J. C. Aronowski J. Savitz S. I. (2011). IL-10 directly protects cortical neurons by activating PI-3 kinase and STAT-3 pathways. *Brain Res.* 1373 189–194. 10.1016/j.brainres.2010.11.096 21138740

[B118] Shelbaya S. Abushady M. M. Nasr M. S. Bekhet M. M. Mageed Y. A. Abbas M. (2017). Study of irisin hormone level in type 2 diabetic patients and patients with diabetic nephropathy. *Curr. Diabetes Rev.* 14 481–486. 10.2174/1573399813666170829163442 28875825

[B119] Solomon V. Goldberg A. L. (1996). Importance of the ATP-ubiquitin-proteasome pathway in the degradation of soluble and myofibrillar proteins in rabbit muscle extracts. *J. Biol. Chem.* 271 26690–26697. 10.1074/jbc.271.43.26690 8900146

[B120] Son J. S. Chae S. A. Testroet E. D. Du M. Jun H. P. (2018). Exercise-induced myokines: a brief review of controversial issues of this decade. *Expert Rev. Endocrinol. Metab.* 13 51–58. 10.1080/17446651.2018.1416290 30063442

[B121] Song G. Ouyang G. Bao S. (2005). The activation of Akt/PKB signaling pathway and cell survival. *J. Cell Mol. Med.* 9 59–71. 10.1111/j.1582-4934.2005.tb00337.x 15784165 PMC6741304

[B122] Sriram S. Subramanian S. Sathiakumar D. Venkatesh R. Salerno M. S. McFarlane C. D. (2011). Modulation of reactive oxygen species in skeletal muscle by myostatin is mediated through NF-kappaB. *Aging Cell* 10 931–948. 10.1111/j.1474-9726.2011.00734.x 21771249 PMC5028794

[B123] Stevens R. G. Graubard B. I. Micozzi M. S. Neriishi K. Blumberg B. S. (1994). Moderate elevation of body iron level and increased risk of cancer occurrence and death. *Int. J. Cancer* 56 364–369. 10.1002/ijc.2910560312 8314323

[B124] Sullivan J. L. (2004). Is stored iron safe? *J. Lab. Clin. Med.* 144 280–284. 10.1016/j.lab.2004.10.006 15614249

[B125] Sunayama J. Tsuruta F. Masuyama N. Gotoh Y. (2005). JNK antagonizes Akt-mediated survival signals by phosphorylating 14-3-3. *J. Cell Biol.* 170 295–304. 10.1083/jcb.200409117 16009721 PMC2171419

[B126] Sutedja N. A. Sinke R. J. Van Vught P. W. Van der Linden M. W. Wokke J. H. Van Duijn C. M. (2007). The association between H63D mutations in HFE and amyotrophic lateral sclerosis in a Dutch population. *Arch. Neurol.* 64 63–67. 10.1001/archneur.64.1.63 17210810

[B127] Tamura Y. Watanabe K. Kantani T. Hayashi J. Ishida N. Kaneki M. (2011). Upregulation of circulating IL-15 by treadmill running in healthy individuals: is IL-15 an endocrine mediator of the beneficial effects of endurance exercise? *Endocr. J.* 58 211–215. 21307608 10.1507/endocrj.k10e-400

[B128] Tasca E. Pegoraro V. Merico A. Angelini C. (2016). Circulating microRNAs as biomarkers of muscle differentiation and atrophy in ALS. *Clin. Neuropathol.* 35 22–30. 10.5414/NP300889 26588026

[B129] Tatemoto K. Hosoya M. Habata Y. Fujii R. Kakegawa T. Zou M. X. (1998). Isolation and characterization of a novel endogenous peptide ligand for the human APJ receptor. *Biochem. Biophys. Res. Commun.* 251 471–476. 10.1006/bbrc.1998.9489 9792798

[B130] Timmons S. Coakley M. F. Moloney A. M. O’Neill C. (2009). Akt signal transduction dysfunction in Parkinson’s disease. *Neurosci. Lett.* 467 30–35. 10.1016/j.neulet.2009.09.055 19800394

[B131] Tisdale M. J. (2004). Cancer cachexia. *Langenbecks Arch. Surg.* 389 299–305. 10.1007/s00423-004-0486-7 15168125

[B132] Torrance J. D. Charlton R. W. Schmaman A. Lynch S. R. Bothwell T. H. (1968). Storage iron in “muscle”. *J. Clin. Pathol.* 21 495–500. 10.1136/jcp.21.4.495 5697351 PMC473840

[B133] Trendelenburg A. U. Meyer A. Rohner D. Boyle J. Hatakeyama S. Glass D. J. (2009). Myostatin reduces Akt/TORC1/p70S6K signaling, inhibiting myoblast differentiation and myotube size. *Am. J. Physiol. Cell Physiol.* 296 C1258–C1270. 10.1152/ajpcell.00105.2009 19357233

[B134] Tsuchiya H. Ebata Y. Sakabe T. Hama S. Kogure K. Shiota G. (2013). High-fat, high-fructose diet induces hepatic iron overload via a hepcidin-independent mechanism prior to the onset of liver steatosis and insulin resistance in mice. *Metabolism* 62 62–69. 10.1016/j.metabol.2012.06.008 22854109

[B135] Vinel C. Lukjanenko L. Batut A. Deleruyelle S. Pradere J. P. Le Gonidec S. (2018). The exerkine apelin reverses age-associated sarcopenia. *Nat. Med.* 24 1360–1371. 10.1038/s41591-018-0131-6 30061698

[B136] Wang Q. Zhang X. Chen S. Zhang S. Youdium M. Le W. (2011). Prevention of motor neuron degeneration by novel iron chelators in SOD1(G93A) transgenic mice of amyotrophic lateral sclerosis. *Neuro Degener. Dis.* 8 310–321. 10.1159/000323469 21346313

[B137] Wang X. Chen W. R. Xing D. (2012). A pathway from JNK through decreased ERK and Akt activities for FOXO3a nuclear translocation in response to UV irradiation. *J. Cell. Physiol.* 227 1168–1178. 10.1002/jcp.22839 21604264

[B138] Wang X. S. Lee S. Simmons Z. Boyer P. Scott K. Liu W. (2004). Increased incidence of the Hfe mutation in amyotrophic lateral sclerosis and related cellular consequences. *J. Neurol. Sci.* 227 27–33. 10.1016/j.jns.2004.08.003 15546588

[B139] Warren S. Torti S. V. Torti F. M. (1993). The role of iron in the cytotoxicity of tumor necrosis factor. *Lymphokine Cytokine Res.* 12 75–80. 8324080

[B140] Wen M. S. Wang C. Y. Lin S. L. Hung K. C. (2013). Decrease in irisin in patients with chronic kidney disease. *PLoS One* 8:e64025. 10.1371/journal.pone.0064025 23667695 PMC3646802

[B141] White M. A. Sreedharan J. (2016). Amyotrophic lateral sclerosis: recent genetic highlights. *Curr. Opin. Neurol.* 29 557–564. 10.1097/WCO.0000000000000367 27538057

[B142] Winkler E. A. Sengillo J. D. Sagare A. P. Zhao Z. Ma Q. Zuniga E. (2014). Blood-spinal cord barrier disruption contributes to early motor-neuron degeneration in ALS-model mice. *Proc. Natl. Acad. Sci. U.S.A.* 111 E1035–E1042. 10.1073/pnas.1401595111 24591593 PMC3964055

[B143] Wong M. Martin L. J. (2010). Skeletal muscle-restricted expression of human SOD1 causes motor neuron degeneration in transgenic mice. *Hum. Mol. Genet.* 19 2284–2302. 10.1093/hmg/ddq106 20223753 PMC2865380

[B144] Wu L. Chen L. Li L. (2017). Apelin/APJ system: a novel promising therapy target for pathological angiogenesis. *Clin. Chim. Acta* 466 78–84. 10.1016/j.cca.2016.12.023 28025030

[B145] Yalcin A. Silay K. Balik A. R. Avcioglu G. Aydin A. S. (2018). The relationship between plasma interleukin-15 levels and sarcopenia in outpatient older people. *Aging Clin. Exp. Res.* 30 783–790. 10.1007/s40520-017-0848-y 29071664

[B146] Yamanaka K. Boillee S. Roberts E. A. Garcia M. L. McAlonis-Downes M. Mikse O. R. (2008). Mutant SOD1 in cell types other than motor neurons and oligodendrocytes accelerates onset of disease in ALS mice. *Proc. Natl. Acad. Sci. U.S.A.* 105 7594–7599. 10.1073/pnas.0802556105 18492803 PMC2396671

[B147] Yasui M. Ota K. Garruto R. M. (1993). Concentrations of zinc and iron in the brains of Guamanian patients with amyotrophic lateral sclerosis and parkinsonism-dementia. *Neurotoxicology* 14 445–450. 8164889

[B148] Zeng X. J. Zhang L. K. Wang H. X. Lu L. Q. Ma L. Q. Tang C. S. (2009). Apelin protects heart against ischemia/reperfusion injury in rat. *Peptides* 30 1144–1152. 10.1016/j.peptides.2009.02.010 19463748

[B149] Zhang J. Guo Y. Wang Y. Song L. Zhang R. Du Y. (2018). Long-term treadmill exercise attenuates Abeta burdens and astrocyte activation in APP/PS1 mouse model of Alzheimer’s disease. *Neurosci. Lett.* 666 70–77. 10.1016/j.neulet.2017.12.025 29246793

[B150] Zhao J. Brault J. J. Schild A. Cao P. Sandri M. Schiaffino S. (2007). FoxO3 coordinately activates protein degradation by the autophagic/lysosomal and proteasomal pathways in atrophying muscle cells. *Cell Metab.* 6 472–483. 10.1016/j.cmet.2007.11.004 18054316

[B151] Zhong S. Xu J. Li P. Tsukamoto H. (2012). Caveosomal oxidative stress causes Src-p21ras activation and lysine 63 TRAF6 protein polyubiquitination in iron-induced M1 hepatic macrophage activation. *J. Biol. Chem.* 287 32078–32084. 10.1074/jbc.M112.377358 22829592 PMC3442538

[B152] Zhong Z. Ilieva H. Hallagan L. Bell R. Singh I. Paquette N. (2009). Activated protein C therapy slows ALS-like disease in mice by transcriptionally inhibiting SOD1 in motor neurons and microglia cells. *J. Clin. Invest.* 119 3437–3449. 10.1172/JCI38476 19841542 PMC2769191

[B153] Zoladz J. A. Pilc A. (2010). The effect of physical activity on the brain derived neurotrophic factor: from animal to human studies. *J. Physiol. Pharmacol.* 61 533–541. 21081796

[B154] Zou Y. Wang B. Fu W. Zhou S. Nie Y. Tian S. (2016). Apelin-13 Protects PC12 cells from corticosterone-induced apoptosis through PI3K and ERKs activation. *Neurochem. Res.* 41 1635–1644. 10.1007/s11064-016-1878-0 26961889

